# Dephosphorylation Passivates the Seeding Activity of Oligomeric Tau Derived From Alzheimer’s Brain

**DOI:** 10.3389/fnmol.2021.631833

**Published:** 2021-05-13

**Authors:** Ruozhen Wu, Longfei Li, Ruirui Shi, Yan Zhou, Nana Jin, Jianlan Gu, Yunn Chyn Tung, Fei Liu, Dandan Chu

**Affiliations:** ^1^NMPA Key Laboratory for Research and Evaluation of Tissue Engineering Technology Products, Key Laboratory of Neuroregeneration of Jiangsu and Ministry of Education, Co-innovation Center of Neuroregeneration, Nantong University, Nantong, China; ^2^Department of Neurochemistry, Inge Grundke-Iqbal Research Floor, New York State Institute for Basic Research in Developmental Disabilities, Staten Island, NY, United States

**Keywords:** Alzheimer’s disease, neurofibrillary tangles, oligomerictau derived from AD brain, dephosphorylation, seedingactivity

## Abstract

Accumulation of intracellular neurofibrillary tangles (NFTs), which are constituted of abnormally phosphorylated tau, is one of the neuropathological hallmarks of Alzheimer’s disease (AD). The oligomeric aggregates of tau in AD brain (AD O-tau) are believed to trigger NFT spreading by seeding normal tau aggregation as toxic seeds, in a prion-like fashion. Here, we revealed the features of AD O-tau by Western blots using antibodies against various epitopes and determined the effect of dephosphorylation on the seeding activity of AD O-tau by capture and seeded aggregation assays. We found that N-terminal truncated and C-terminalhyperphosphorylated tau species were enriched in AD O-tau. Dephosphorylation of AD O-tau by alkaline phosphatasediminished its activity in capturing tau *in vitro* and ininducing insoluble aggregates in cultured cells. Our resultssuggested that dephosphorylation passivated the seeding activity ofAD O-tau. Inhibition of phosphorylation may be a potentstrategy to prevent the spreading of tau patho3logy.

## Introduction

The microtubule-associated protein tau is a highlywater-soluble and basic protein. Normal tau stabilizes microtubules *in vitro* by binding tothe interface between tubulin heterodimers with itsmicrotubule-binding repeats (Kadavath et al., 2015). As a phosphoprotein, tau contains more than 80 residues that can potentially bephosphorylated, and at least 18 of these sites are abnormallyhyperphosphorylated in the brains of Alzheimer’s patients(Chu and Liu, 2018; Iqbal et al., 2018). Hyperphosphorylated tau detachesfrom microtubules, resulting in microtubule loss in neurons(Austin et al., 2017). Accumulation of intracellular neurofibrillarytangles (NFTs), which mainly consist of hyperphosphorylated andtruncated tau, is correlated directly with the degree of cognitivedecline in AD patients and is considered as one of the predominanthallmarks of AD.

The NFT pathology in AD brains initiates in the locus coeruleus and transentorhinal area, and sequentially progresses to the limbic system and further to the isocortex, as described in the Braak stages (Braak and Braak, 1991). Intrahippocampal injection of tau aggregates isolated from AD patients or produced *in vitro* successfully induced tau hyperphosphorylation and NFT formation at the injection sites and anatomically related regions in rodent brains, showing a similar stereotypical propagation of tau pathology as observed in AD brain (Clavaguera et al., 2009; Boluda et al., 2015; Takeda et al., 2015; Hu et al., 2016; Miao et al., 2019). Emerging evidence suggests that the prion-like seeding activity of pathological tau in AD brain is crucial for its propagation. Due to the induction of molecules with strong negative charges (such as heparin and RNA) or even proteins, the inert tau monomer could switch its conformation to form β-sheet structures that are prone to oligomerization (Goedert et al., 1996; Mudher et al., 2017; Wischik et al., 2018). The oligomeric tau aggregates, acting like “seeds,” capture normal tau proteins and template their conformational change in a prion-like mechanism, and eventually assembles the paired helical filaments (PHFs) and NFTs in neurons (von Bergen et al., 2005; Lasagna-Reeves et al., 2012b). Oligomeric tau isolated from Alzheimer’s brain (AD O-tau) has been reported to capture tau protein *in vitro*, seed tau aggregation in cultured cells, and induce the propagation of tau pathology in rodent brains (Lasagna-Reeves et al., 2012a; Hu et al., 2016; Li et al., 2019).

Our previous studies showed that high-molecular-weightoligomers of tau (HMW-tau) were specifically accumulated in thebrain of AD patients. HMW-tau lacked the N-terminal portionand was hyperphosphorylated at multiple sites including Ser199, Ser202, Thr205, Thr212, Ser214, Thr217, Ser262, Ser396, Ser404, andSer422 (Zhou et al., 2018; Li et al., 2019). According to the reportedcleavage sites on human tau in AD brain (Wang et al., 2010; Zhang et al., 2014), we deleted the first 50, 150 or 230 amino acids(a.a.) and the last 20 or 50 a.a. of the longest human tau isoformtau441, examined the pathological activities of each truncatedisoforms, and found that deletion of the first 150 and the last 50 a.a. of tau enhanced its site-specific phosphorylation, self-aggregation, and captured and seeded aggregation by ADO-Tau (Gu et al., 2020).

Dephosphorylation of AD hyperphosphorylated tau with proteinphosphatases such as protein phosphatase 2A (PP2A) restored themicrotubule polymerization activity of tau (Wang et al., 1996). Dephosphorylation with alkaline phosphatase (AP) or PP2A diminishedthe ability of hyperphosphorylated tau in inducing aggregation, prevented tangle formation *in vitro* (Alonso et al., 1996), andreduced the number of NFTs in mouse brain (Hu et al., 2016). However, the mechanism that dephosphorylation inhibits the prion-likeactivity of toxic tau seeds remains unclear.

In the present study, we isolated oligomeric tau from AD brains and analyzed AD O-tau by Western blots using antibodies against different epitopes of tau protein. We found that AD O-tau was mainly N-terminal truncated and C-terminal hyperphosphorylated. AP treatment successfully reduced the phosphorylation of AD O-tau. Tau capture assay revealed that compared with AD O-tau, the ability of dephosphorylated AD O-tau (Dp-AD O-tau) to capture free tau is decreased. Immunofluorescence showed that Dp-AD O-tau templated less aggregates formation in HeLa cells. Seeded tau aggregation assay in HEK-293FT cells revealed that Dp-AD O-tau induced less accumulation of total and phosphorylated tau in the insoluble fractions from cell lysates. Our results suggested that dephosphorylation could be an effective way to passivate the prion-like seeding activity of AD O-tau.

## Materials and Methods

### Human Brain Samples

Frozen frontal cortices from autopsied and histopathologicallyconfirmed AD (80 years old, female, 2.9 h post mortem interval, Harvard Brain Tissue Resource Center McLean Hospital) andage-matched normal human (84 years old, female, 4.25 h post morteminterval, De Nederlandse Hersenbank) brains were obtained withoutidentification of donors. Brain samples were frozen at−80°C until analysis. The use of postmortem human braintissue was in accordance with the National Institutes of HealthGuidelines and was exempted by the Institutional Review Board ofNew York State Institute for Basic Research in DevelopmentalDisabilities because “the research does not involve intervention orinteraction with the individuals,” nor “is the informationindividually identifiable.”

### Preparation of AD O-Tau, Dephosphorylated-AD O-Tau (Dp-AD O-Tau), and Heat-Stable Tau (HS-Tau)

AD O-tau was isolated from frozen autopsy cerebral cortex of AD patient as described (Kopke et al., 1993). Briefly, the brain tissue was homogenized in ninefold volume of ice-cold lysis buffer containing 20 mM Tris-HCl, pH 8.0, 0.32 M sucrose, 10 mM β-mercaptoethanol, 10 mM glycerophosphate, 5 mM MgSO_4_, 50 mM NaF, 1 mM EDTA, 1 mM Na_3_VO_4_, 1 mM 4-(2-aminoethyl) benzenesulfonyl fluoride hydrochloride (AEBSF), and 10 μg/ml each of aprotinin, leupeptin, and pepstatin. The homogenate was centrifuged at 27,000 × *g* for 30 min at 4°C. The supernatant was aspirated and further centrifuged at 235,000 × *g* for 45 min at 4°C. The resulting pellet rich in AD O-tau was collected, washed twice, and resuspended in saline. AD O-Tau was stored at −80°C and probe-sonicated for 2 min (0.5 s on, 3 s off) at 20% power before use.

The supernatant was saved and NaCl was added to a final concentration of 0.75 M. The remaining aggregated tau in the supernatant was removed by boiling for 5 min. After cooling, the samples were centrifuged at 25,000 × *g* for 30 min. The supernatant was collected, dialyzed against 10 mM Tris-HCl, pH 7.6, and concentrated by five times to obtain HS-tau.

Dp-AD O-tau was obtained by incubating AD O-tau (4 mg/ml protein) with 196 U/ml AP in the reaction buffer (100 mM Tris-HCl, pH 8.0, 1 mM phenylmethylsulfonyl fluoride, 2 μg/ml aprotinin, 2 μg/ml pepstatin, and 5 μg/ml leupeptin) at 37°C for 5 h. Dephosphorylated products were analyzed by Western blots developed with Tau-1 antibody to show dephosphorylation efficacy.

### Dephosphorylation of AD O-Tau on Nitrocellulose Membrane

Various amounts of AD O-tau were spotted on a nitrocellulosemembrane (Schleicher and Schuell, Keene, NH, United States) at 5 μl/grid of 7 × 7 mm. The membrane was incubated at37°C for 1 h to allow protein binding to the membrane. Proteins on the membrane were dephosphorylated as describedpreviously (Kopke et al., 1993) with slight modification. The membranewas wet by TBS and incubated with 196 U/ml AP in the reaction bufferas described above at 37°C for 5 h. After blocking with 5% milk in TBS (50 mM Tris-HCl, pH 7.4, 150 mM NaCl) for 30 min, membrane was then developed with specific antibodies or subjected totau capture assay.

### Western Blots and Dot Blots

Brain homogenates were prepared in 10% in ice-cold lysis buffer consisting of 50 mM Tris-HCl, pH 7.4, 8.5% sucrose, 2.0 mM EDTA, 10 mM β-mercaptoethanol, 1 mM Na_3_VO_4_, 50 mM NaF, 1 mM AEBSF, and 10 μg/ml each of aprotinin, leupeptin, and pepstatin. The homogenates were diluted in 2 × Laemmli sample buffer (250 mM Tris-HCl, pH 6.8, 20% glycerol, 20% β-mercaptoethanol, 4% SDS and 0.008% bromophenol blue), followed by boiling for 10 min. Protein concentration of each sample was measured by the Pierce^TM^ 660 nm Protein Assay kit (Thermo Fisher Scientific, Waltham, MA, United States). Same amounts of protein in brain homogenate were subjected to sodium dodecyl sulfate-polyacrylamide gel electrophoresis (SDS-PAGE) and electro-transferred to a polyvinylidene difluoride (PVDF) membrane.

For dot blots, the cells were lysed with RIPA (radio immunoprecipitation assay) buffer (50 mM Tris-HCl, pH 7.4, 150 mM NaCl, 1% Non-idet P-40, 0.5% sodium deoxycholate, 0.1% SDS, 50 mM NaF, 1 mM Na_3_VO_4_, 1 mM AEBSF, 5 mM benzamidine, and 10 μg/ml each of aprotinin, leupeptin, and pepstatin) and the protein concentration was determined. Different amounts of protein were spotted on a nitrocellulose membrane (Schleicher and Schuell, Keene, NH, United States) at 5 μl/grid of 7 × 7 mm. The blot was incubated at 37°C for 1 h to allow protein binding to the membrane.

The PVDF or nitrocellulose membrane was blocked with 5% skim milkin TBS buffer (50 mM Tris-HCl, pH 7.4, and 150 mM NaCl) for 30 minand was subsequently incubated overnight with specific primaryantibodies (Table 1) at room temperature. After washing three times with TBST (TBS containing 0.05% Tween-20), the membrane was incubated with horseradish peroxidase(HRP)-conjugated secondary antibody (1:4,000, JacksonImmunoResearch, West Grove, PA, United States) at room temperaturefor 2 h, washed with TBST, incubated with enhanced chemiluminescence(ECL) kit (Thermo Fisher Scientific, Rockford, IL, United States), and exposed to X-ray films (Denville Science, Holliston, MA, United States). The intensity of bands on Western blots and dotblots was quantified by Multi Gauge software (Fujifilm, Mito, Tokyo, Japan).

**TABLE 1:** Primary antibodies used in this study.

**Antibody**	**Type**	**Specificity**	**Working dilution**	**Species**	**Source (catalog number)**
113e	Polyclonal	Pan-tau (a.a. 19–32)	1:1,000	Rabbit	In-house
43D	Monoclonal	Pan-tau (a.a. 6–18)	1:1,000	Mouse	BioLegend (816601)
77G7	Monoclonal	Pan tau (a.a. 244–368)	1:1,000	Mouse	BioLegend (816701)
92e	Polyclonal	Pan-tau	1:1,000	Rabbit	In-house
Anti-HA	Polyclonal	HA	1:2,000	Rabbit	Sigma (H6908)
Anti-HA	Monoclonal	HA	1:30,000	Mouse	Sigma (H9658)
Anti-pT181	Monoclonal	Phospho-tau (T181)	1:500	Mouse	Invitrogen (MN1050)
Anti-pS199	Polyclonal	Phospho-tau (S199)	1:1,000	Rabbit	Invitrogen (44-734G)
Anti-pT212	Polyclonal	Phospho-tau (T212)	1:500	Rabbit	Invitrogen (44-740G)
Anti-pS214	Polyclonal	Phospho-tau (T214)	1:1,000	Rabbit	Invitrogen (44-742G)
Anti-pT217	Polyclonal	Phospho-tau (T217)	1:500	Rabbit	Invitrogen (44-744)
Anti-pS262	Polyclonal	Phospho-tau (S262)	1:500	Rabbit	Invitrogen (44-750G)
Anti-pS422	Polyclonal	Phospho-tau (S422)	1:500	Rabbit	Invitrogen (44-764G)
HT7	Monoclonal	Pan-tau (a.a. 159–163)	1:1,000	Mouse	Invitrogen (MN1000)
PHF-1	Monoclonal	Phospho-tau (S396/404)	1:500	Mouse	Dr. Peter Davies
R134d	Polyclonal	Pan-tau	1:10,000	Rabbit	In-house
Tau-1	Monoclonal	Unphosphorylated-tau (a.a. 195,198,199, and 202)	1:500	Mouse	Dr. Lester I. Binder
Tau46	Monoclonal	Pan tau (a.a. 404–421)	1:1,000	Mouse	Invitrogen (13-6400)
TAU-5	Monoclonal	Pan-tau (a.a. 210–230)	1:1,000	Mouse	Invitrogen (AHB0042)
GAPDH	Monoclonal	GAPDH	1:1,000	Mouse	Santa Cruz (sc-47724)

### Cell Culture and Plasmids

HEK-293FT and HeLa cell lines were maintained in Dulbecco’s modified Eagle’s medium (DMEM) supplemented with 10% fetal bovine serum (Thermo Fisher Scientific, Waltham, MA, United States) at 37°C in a humidified atmosphere of 5% CO_2_/95% air.

pCI/HA (hemagglutinin)-tau_1–4__41_ andpCI/HA-tau_15__1–3__91_ were generated as previouslydescribed (Gu et al., 2020). The DNA sequence of the plasmids wasconfirmed by Sanger sequencing. PCI-neo plasmid was used as anegative control. pCI/HA-tau_1–4__41_ orpCI/HA-tau_15__1–3__91_ was transfected intoHEK-293FT or HeLa cells by FuGENE^®^, HD TransfectionReagent (Promega, Madison, WI, United States) following themanufacturer’s instructions.

### Seeded Tau Aggregation in Cultured Cells

HEK-293FT cells were transfected with pCI/HA-tau_1–4__41_ or pCI/HA-tau_15__1–3__91_. AD O-Tau or Dp-AD O-tau was sonicated at 20% power for 2 min (0.5 s on, 3 s off) before use. Six hours after transfection, AD O-Tau or Dp-AD O-tau was mixed with 3% Lipofectamine 2000 (Invitrogen, Carlsbad, CA, United States) in Opti-MEM (Thermo Fisher Scientific, Waltham, MA, United States) for 20 min at room temperature and then added into the cell culture at a final concentration of 6.6 μg/ml. Forty-eight hours after transfection, the cells were harvested and analyzed for tau aggregation by Western blots or immunofluorescence.

To examine the accumulation of insoluble tau, the cells were lysed in RIPA buffer for 20 min on ice and centrifuged at 130,000 × *g* for 45 min. The supernatants were collected as RIPA-soluble fraction and diluted in 4 × Laemmli sample buffer. The resulting pellets containing the insoluble fraction were washed twice with RIPA buffer and resuspended in 1 × Laemmli buffer by sonicating at 80% power for 8 min (5 s on, 20 s off) with sonicator equipped with cup horn (Thermo Fisher Scientific, Waltham, MA, United States). The samples were boiled for 5 min. Levels of RIPA-insoluble and -soluble tau were determined by Western blots developed with anti-HA antibody (Table 1). Levels of phosphorylated tau at each specific site were analyzed by Western blots developed with site-specific and phosphorylation-dependent tau antibodies.

### Immunofluorescence

HeLa cells were transfected with pCI/HA-tau_1–4__41_ orpCI/HA-tau_15__1–3__91_ by FuGENE^®^, HDand treated with 6.6 μg/ml AD O-tau or Dp-ADO-tau as described above. The cells were harvested, fixed for15 min with 4% paraformaldehyde in phosphate-buffered saline (PBS), and treated with 0.3% Triton in PBS for 15 min. After blocking with5% newborn goat serum, 0.05% Tween-20, and 0.1% Triton X-100 inPBS for 30 min, the cells were incubated with 77G7 (1:500) oranti-HA (1:1,000) antibody in the blocking solution overnight at4°C. After washing three times with PBS, the cells wereincubated with Alexa Fluor 555-conjugated or 488-conjugated IgG(1:1,000, Thermo Fisher Scientific, Waltham, MA, United States) for2 h and TO-PRO-3 (5 mg/ml, Thermo Fisher Scientific, Waltham, MA, United States) for 15 min at room temperature. After washing withPBS, the cells were mounted using ProLong^TM^ GoldAntifade Mountant (Thermo Fisher Scientific, Waltham, MA, United States). The images were captured with a Nikon EZ-C1 confocalmicroscope (Nikon Instruments, Melville, NY, United States).

The number of HA-tau_15__1–3__91_-expressing cells and the number of cells with tau aggregates were counted under the microscope. The percentage of cells with tau aggregates was calculated by dividing the number of cells with aggregates by the total number of HA-tau_15__1–3__91_-expressing cells on each slide.

### Tau Capture Assay

Tau capture assay was carried out as described (Kremer et al., 1988; Alonso et al., 1994; Li et al., 2019). HEK-293FT cells overexpressingpCI/HA-tau_15__1–3__91_ were lysed in PBS containing50 mM NaF, 1 mM Na_3_VO_4_, 1 mM AEBSF, 5 mM benzamidine, and 10 μg/ml each of aprotinin, leuprotinin, and pepstatin. The cell lysate was sonicated at 20% power for 2 min (0.5 s on, 3 soff) and centrifuged at 10,000 × *g* for 10 min toextract the supernatant containingHA-tau_15__1–3__91_. Different amounts of ADO-tau or HS-tau were spotted on the nitrocellulose membraneand dried at 37°C for 1 h. The membrane was then blockedwith 5% skim milk in TBS for 1 h, and incubated overnight with thecell extract containing HA-tau_15__1–3__91_. Afterwashing three times with TBST, the membrane was examined by anti-HAantibody using dot blot method.

### Statistical Analysis

Data were presented as mean ± standard deviation (SD). The statistical significance was analyzed by one-way analysis of variance (ANOVA) followed by Tukey’s *post hoc* test for multiple comparisons using GraphPad Prism 8 (GraphPad Software Inc., San Diego, CA, United States). *P* < 0.05 was considered statistically significant.

## Results

### AD O-Tau Is Mainly N-Terminal Truncated and C-Terminal Hyperphosphorylated

**FIGURE 1:**
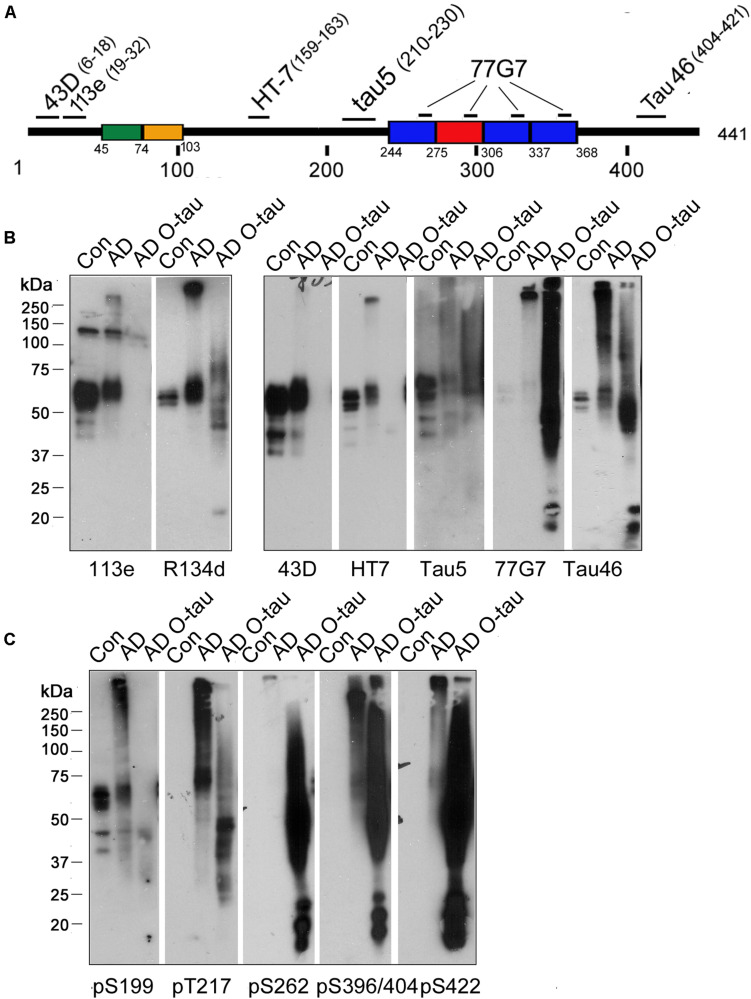
ADO-tau wasN-terminal truncated C-terminal hyperphosphorylated. **(A)** The diagram of the longest human tau isoform(tau_441_) showing the epitopes recognized by various pan-tauantibodies used in this study. **(B,C)** AD O-tau andthe homogenates from AD and control human brains were analyzed byWestern blots developed with polyclonal or monoclonal pan-tauantibodies **(B)** and site-specific andphosphorylation-dependent tau antibodies **(C)**.

Truncation and hyperphosphorylation of tau are commonly found in ADbrains (Zhou et al., 2018; Li et al., 2019). Brain-derived tau oligomersfrom the individuals with AD and related tauopathies can capturenormal tau and trigger the propagation of tau pathology as cytotoxicseeds (Hu et al., 2016; Chu and Liu, 2018). To determine whetheroligomeric tau from AD brain is also hyperphosphorylated and/ortruncated, AD O-tau was analyzed by Western blots developedwith antibodies raised against specific epitopes of tau (Figure 1A and Table 1). R134d, a polyclonal antibodyraised against the longest human tau isoform, tau441 (Tatebayashi et al., 1999), detected smear bands in AD O-tau and ADbrain lysate but not in control brain lysates, confirming thetruncation and SDS- and β-mercaptoethanol-resistanthigh-molecular weight of AD O-tau (Figure 1B). Monoclonal antibodies 43D andHT7, which recognize the N-terminal 6–18 a.a. and 159–163 a.a. of tau441, respectively, detected tau bands in both control andAD brain lysates, but not in AD O-tau. Similar results werefound when using the polyclonal antibody 113e (Li et al., 2019) thatrecognizes 19–32 a.a. of tau, indicating that AD O-tau wasmostly N-terminal truncated (Figure 1B). 77G7 (244–368 a.a.) and Tau5 (210–230 a.a.) that target the epitopes in or close to themicrotubule binding repeats of tau both showed stronger affinity toAD O-tau than to AD or control brain lysates. The level oftau detected by Tau46 (404–421 a.a.) was decreased in ADO-tau, implying that AD O-tau could be partiallyC-terminal truncated. These results suggested that truncatedtau species were accumulated in AD O-tau.

Tau is abnormally hyperphosphorylated in AD brain (Zhou et al., 2018). Therefore, we used a series of phosphorylation site-specific antibodies of tau to examine the phosphorylation pattern of AD O-tau (Figure 1C). Phosphorylation of all the epitopes tested, pS199, pT217, pS262, pS396/404, and pS422, were increased in AD compared to control lysate, consistent with previous reports (Zhou et al., 2018). However, the changes of site-specific phosphorylation in AD O-tau were not the same as that in AD lysate. In AD O-tau, pS199 phosphorylation was distinctly eliminated, which might be because of tau cleavage at the N-terminus. pT217 showed comparable phosphorylation levels in AD O-tau and AD lysate. Phosphorylation of pS262, pS396/404, and pS422 was dramatically elevated in AD O-tau than in AD and control lysate. The site-selective phosphorylation of AD O-tau implied that C-terminal hyperphosphorylated tau species were prone to aggregate in AD O-tau.

### Dephosphorylation Inhibits the Ability of AD O-Tau to Capture Tau_15__1__–3__91_

**FIGURE 2:**
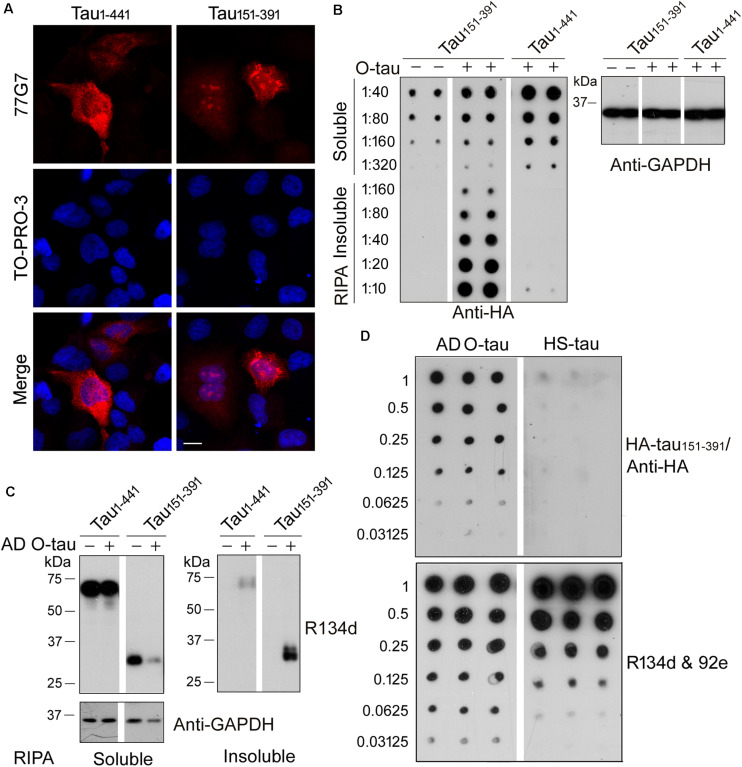
AD O-tau captured HA-tau_15__1–3__91_ *in vitro* and seeded its aggregation in cultured cells. **(A)** HeLa cells were transfected with pCI/HA-tau_1–4__41_ or pCI/HA-tau_15__1–3__91_, treated with AD O-tau for 42 h and immunostained with tau antibody 77G7 (red). Cell nuclei were labeled by TO-PRO-3 (blue). Bar, 10 μm. **(B,C)** HEK-293FT cells were transfected with pCI/HA-tau_1–4__41_ or pCI/HA-tau_15__1–3__91_, treated with AD O-tau for 42 h and lysed with RIPA buffer. RIPA buffer-soluble and -insoluble fractions were separated by centrifuging at 130,000 × *g* for 45 min. Dilution series of RIPA-soluble or -insoluble samples were applied on nitrocellulose membranes and detected with anti-HA antibody. Corresponding cell lysates from parallel experiment were analyzed by Western blot developed with anti-GAPDH **(B)**. RIPA-soluble or -insoluble samples were analyzed by Western blots developed with anti-tau (R134d and 92e) and anti-GAPDH antibodies **(C)**. **(D)** A series of concentration gradients of AD O-tau or heat-stable tau (HS-tau) were applied on nitrocellulose membranes. The membrane was incubated with HEK-293FT cell lysate expressing HA-tau_15__1–3__91_. HA-tau_15__1–3__91_ captured by AD O-tau or HS-tau was detected with anti-HA antibody. The total amount of AD O-tau or HS-tau on the membrane was determined by the mixture of pan-tau antibodies, R134d and 92e.

Recently, we constructed 11 truncated forms offull-length tau and analyzed the pathological activities of them(Gu et al., 2020). We found that deletion of the first 150 or230 a.a. and the last 50 a.a. of tau enhanced its site-specificphosphorylation and seeded aggregation by AD O-Tau. Amongthese truncated proteins, tau_15__1–3__91_ exhibitedthe highest pathological activities but did not show significantdifference in cytotoxicity compared with full-length tau(tau_1–4__41_) (Gu et al., 2020). In the present study, HeLa cells were transfected with HA-tau_1–4__41_ orHA-tau_15__1–3__91_. Six hours later, the cells weretreated with AD O-tau for 42 h and then subjected toimmunofluorescence. The immunofluorescence staining with 77G7 antibodies revealed that both HA-tau_1–4__41_ andHA-tau_15__1–3__91_ were expressed in HeLa cells. ADO-tau induced remarkable aggregates in the cytoplasm andnucleus of HA-tau_15__1–3__91_-expressing cells, butonly diffusion distribution of tau inHA-tau_1–4__41_-transfectedcells (Figure 2A).

For biochemical analyses, HEK-293FT cells were transfected with HA-tau_1–4__41_ or HA-tau_15__1–3__91_, treated with AD O-tau for 42 h and then lysed with RIPA buffer and centrifuged at 130,000 × *g* for 45 min to separate RIPA-insoluble pellets and RIPA-soluble supernatants. Serial dilutions of the RIPA-soluble or -insoluble samples were spotted on nitrocellulose membranes and subjected to dot blots using anti-HA antibody (Figure 2B). The results showed higher level of RIPA-soluble tau in HEK-293FT/HA-tau_1–4__41_ cells than in HEK-293FT/HA-tau_15__1–3__91_ cells. O-tau treatment induced intensive insoluble aggregates of HA-tau_15__1–3__91_, but not that of HA-tau_1–4__41_. Next, RIPA-soluble or -insoluble fraction from HA-tau_1–4__41_- or HA-tau_15__1–3__91_-expressing cells treated with or without AD O-tau were examined by Western blots developed with tau antibodies (Figure 2C). AD O-tau induced dramatic accumulation of tau_15__1–3__91_ in RIPA-insoluble fraction, whereas less tau_15__1–3__91_ in RIPA-soluble fraction, compared with tau_1–4__41_. These results further support that tau_15__1–3__91_ could be more susceptible to AD O-tau seeded aggregation. Thus, HA-tau_15__1–3__91_, instead of HA-tau_1–4__41_, was used in the following analyses for tau seeding activity.

Normal tau is heat-stable (Weingarten et al., 1975). Heat treatment iscommonly used to remove tau aggregates from the soluble fraction ofbrain lysate (Planel et al., 2007; Miao et al., 2019). Various amounts of ADO-tau or heat-stable tau (HS-tau) were dotted onnitrocellulose membranes, which were further incubated with celllysate containing HA-tau_15__1–3__91_ to allow thecapture of HA-tau_15__1–3__91_ by AD O-tau orHS-tau. Captured HA-tau_15__1–3__91_ was detected byanti-HA antibody (Figure 2D). The resultsindicated that AD O-tau, but not HS-tau, capturedHA-tau_15__1–3__91_ (Figure 2D).

**FIGURE 3:**
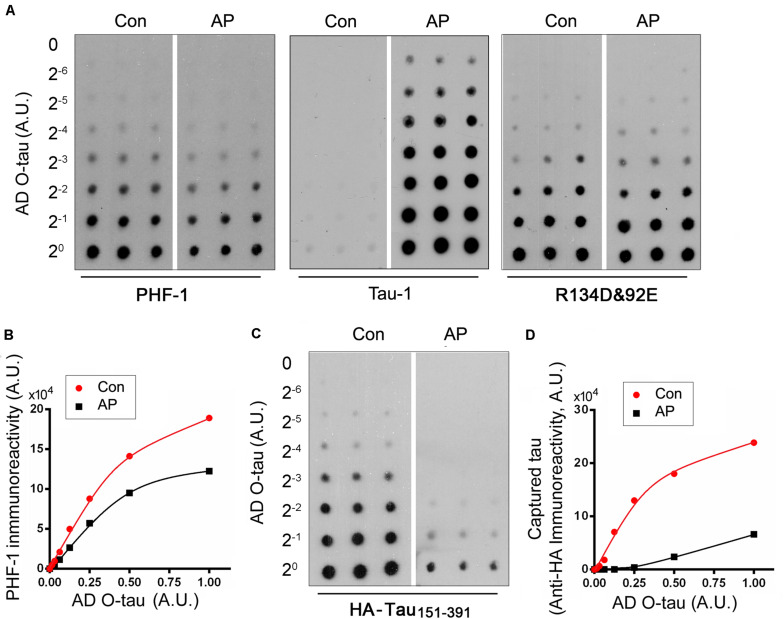
Dephosphorylation of AD O-tau inhibited its ability to capture HA-tau_15__1–3__91_ *in vitro*. **(A,B)** A series of concentration gradients of AD O-tau were applied onto nitrocellulose membranes. One set of membranes were treated with alkaline phosphatase (AP) to dephosphorylate AD O-tau. The control membranes were incubated with only the reaction buffer. The membranes were then developed with PHF-1 (anti-pS396/404 tau) or a mixture of R134d and 92e (pan-tau) to analyze the phosphorylation and total levels of AD O-tau, respectively **(A)**. The relative levels of PHF-1 immunoreactivity were plotted against the amounts of AD O-tau or Dp-AD O-tau **(B)**. **(C,D)** Control or AP-treated membranes were incubated with HEK-293FT cell lysate expressing HA-tau_15__1–3__91_ and developed with anti-HA antibody to measure the levels of HA-tau_15__1–3__91_ captured by AD O-tau or Dp-AD O-tau **(C)**. Anti-HA immunoreactivity was plotted against the amounts of AD O-tau and Dp-AD O-tau **(D)**.

To determine the role of phosphorylation in modulating the captureability of AD O-tau, we applied various amount of ADO-tau onto nitrocellulose membranes parallelly. One set ofmembranes was treated with AP to dephosphorylate AD O-tau onit. The control membranes were treated with the reaction bufferparallelly. Dephosphorylation of AD O-tau by AP wasidentified by reduced PHF-1 (recognizing phosphorylated tau atSer396/404) and enhanced Tau-1 (recognizing tau unphosphorylatedSer195/198/199/202) immunoreactivity(Figures 3A,B). Similar R134d(Tatebayashi et al., 1999) and 92e (Grundke-Iqbal et al., 1988) immunoreactivity indicatedthat AP treatment did not affect total tau levels on the membrane(Figure 3A). The control and AP-treatedmembranes were further incubated with cell lysate containingHA-tau_15__1–3__91_ to allow the binding ofHA-tau_15__1–3__91_ to AD O-tau. CapturedHA-tau_15__1–3__91_ developed with anti-HA wasmarkedly decreased in AP-treated membrane, suggesting thatdephosphorylation of AD O-tau inhibited its ability tocapture tau (Figures 3C,D).

### Dephosphorylation Inhibits AD O-Tau-Induced Aggregation of Tau_15__1__–3__91_

**FIGURE 4:**
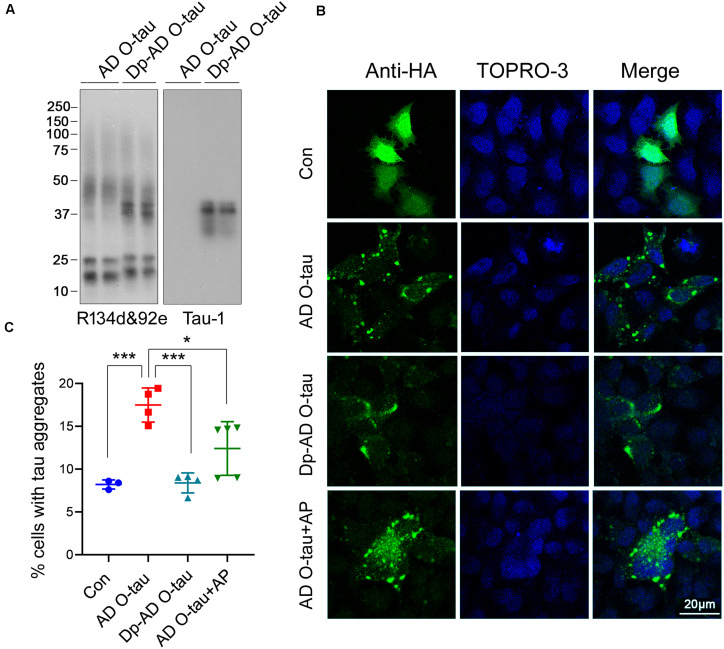
Dephosphorylation of AD O-tau inhibited its abilityto seed tau aggregation in HeLa cells. **(A)** AD O-tauand Dp-AD O-tau were analyzed by Western blots developed witha mixture of R134d and 92e (pan-tau) or Tau-1 (unphosphorylatedtau). **(B,C)** HeLa cells were transfected withpCI/HA-tau_15__1–3__91_, treated with ADO-tau, Dp-AD-O-tau, or AD O-tau and AP for 42 h, andimmunostained with anti-HA antibody (**B**, green). Cell nucleiwere labeled by TO-PRO-3 (**B**, blue). Bar, 20 μm. Thenumber of HA-tau_15__1–3__91_-expressing cells andthe number of cells with tau aggregates were counted **(C)**. The experiment was performed in triplicate wells and eight fieldswere photographed from each well of each group. Percentage of cellswith aggregated tau was determined. Each experiment was repeated atleast twice. *P < 0.05. ***P < 0.001.

To assess the ability of dephosphorylated AD O-tau (Dp-ADO-tau) in triggering tau aggregation, we used AP todephosphorylate AD O-tau and added Dp-AD O-tau intoHeLa cells expressing HA-tau_15__1–3__91_ toinvestigate the formation of aggregates. Western blots developedwith Tau-1 antibody (Binder et al., 1985) confirmed the up-regulation ofdephosphorylation levels in Dp-AD O-tau after AP treatment(Figure 4A). Overexpression ofHA-tau_15__1–3__91_ in HeLa cells usually leads toaggregation in less than 10% of the transfected cells (HA positive) after 48 h transfection when examined by immunofluorescence(Figure 4C). Incubation with AD O-taufor 42 h significantly increased the proportion of aggregate-bearingcells (Figures 4B,C; Li et al., 2019; Gu et al., 2020). Unlike AD O-tau, Dp-AD O-tau did notpromote the formation of tau aggregates(Figures 4B,C). In another set ofexperiments, 0.26 U AP was added into the culture medium ofHA-tau_15__1–3__91_-expressing cells together with ADO-tau and also inhibited AD O-tau-induced tauaggregation (Figures 4B,C). These resultssuggested that dephosphorylation of AD O-tau and/or itsrecruiting targets could help to reduce the formationof tau aggregates.

**FIGURE 5:**
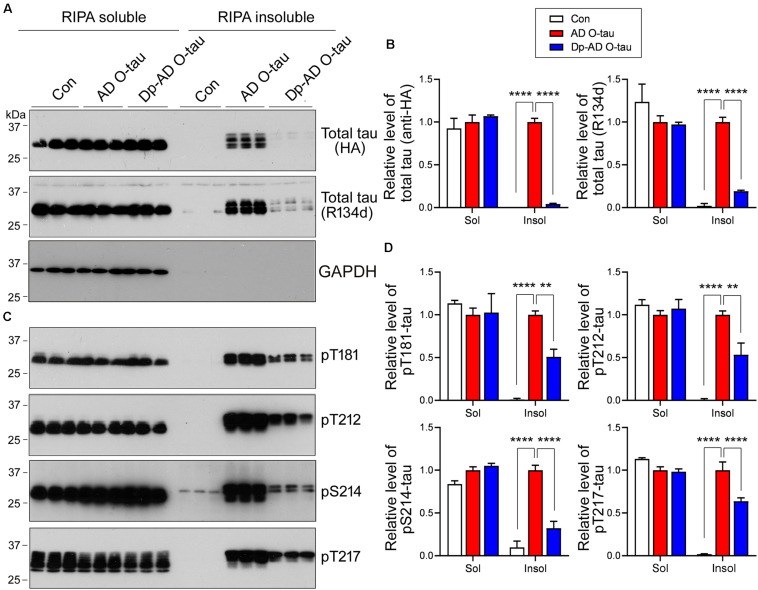
Dephosphorylation of AD O-tau suppressed itsability to template tau aggregation. **(A–D)** HEK-293FT cellswere transfected with pCI/HA-tau_15__1–3__91_, treated with AD O-tau or Dp-AD O-tau and lysed withRIPA buffer. RIPA-soluble and-insoluble fractions were separated bycentrifugation and analyzed with Western blots developed withantibodies toward HA, total tau (R134d), GAPDH **(A)**, orvarious phosphorylated tau **(C)**. The experiment wasperformed in triplicate. Relative levels of total **(B)** orphosphorylated **(D)** tau were measured. ^∗∗^*P* < 0.01. ^****^P < 0.0001.

Next, HEK-293FT cells were transfected with HA-tau_15__1–3__91_ and treated with AD O-tau or Dp-AD O-tau. The cell lysates were separated into RIPA-soluble and RIPA-insoluble fractions. Total and phosphorylated tau levels in each fraction were analyzed by Western blot (Figure 5). AD O-tau induced dramatic accumulation of total tau (Figures 5A,B) and tau phosphorylated at T181, T212, S214, and T217 (Figures 5C,D) in RIPA-insoluble fractions. However, compared with AD O-tau, Dp-AD O-tau treatment significantly decreased both total and phosphorylated tau deposit in RIPA-insoluble fractions (Figure 5). In both groups, the levels of RIPA-soluble tau were not grossly affected. These data further supported that dephosphorylation of AD O-tau could suppress its ability in templating tau aggregation.

## Discussion

Oligomeric tau derived from Alzheimer’s brain is capable of capturing normal tau and templating misfolding and aggregation of tau protein and is therefore considered to display prion-like seeding properties. However, the characteristics of AD O-tau were not fully understood. Here, we reported that AD O-tau was mainly N-terminal truncated and C-terminal hyperphosphorylated. Dephosphorylation of AD O-tau significantly blocked its ability to capture tau and template tau aggregation.

NFTs composed of abnormally hyperphosphorylated tau are majorpathological hallmarks of AD (Iqbal et al., 2016). Normal tau containstwo to three phosphate groups. However, the phosphorylation levelsof tau are increased by two to three times in AD brains. Hyperphosphorylation of tau alters the protein charge andconformation, which makes it easier to aggregate (Iqbal et al., 2016). Tau in AD brain displays in various pools—cytosolic and normal tau(AD-tau), cytosolic and hyperphosphorylated/oligomeric tau (ADP-tau), and PHF-tau (Kopke et al., 1993). AD P-tau, butnot PHF tau, sequesters/captures normal tau *in vitro* toform filaments in a non-saturable manner (Alonso et al., 1994), which wasthe first identification of prion-like activity of AD P-tau. AD P-tau was isolated from the 27,000 to200,000 × *g* fraction of AD brain homogenate byextraction in 8 M urea and was further purified by acidprecipitation and ion chromatography (Kopke et al., 1993). It was foundthat 3,000 and 10,000 × *g* AD brain extracts, whichpresumably contained HMW proteins, showed significantly higherseeding activity than 150,000 × *g* extracts fromwhich HMW tau was depleted by sedimentation (Takeda et al., 2015). The3,000 and 10,000 × *g* extracts contain various sizesof oligomeric tau (Takeda et al., 2015). Thus, AD O-tau in thefraction by sedimentation of AD brain homogenates from 27,000 to235,000 × *g* predominantly varies in size ofoligomeric tau, which is hyperphosphorylated and displays SDS- andβ-mercaptoethanol-resistant HMW-tau. However, the sizedistribution of AD O-tau remains to be characterized.

Recently, studies utilizing high-resolution quantitative proteomics (Wesseling et al., 2020) and mass spectrometry (Dujardin et al., 2020) identified 26 post-translational modifications (PTM) in HMW-tau oligomers. Although tau PTM profiles were heterogeneous across subjects, the peptides spanning amino acid residues 195–209, 212–224, and 396–406 featured high-frequency phosphorylation and showed > 90% modification extent in AD patients (Wesseling et al., 2020). Phosphorylation on Ser262 was positively correlated with tau seeding capacity (Dujardin et al., 2020). Consistent with these studies, pT217, pSer262, and pS396/404 were dramatically elevated in AD O-tau compared to the control in this study. Ser422, which is not considered as a phosphorylation hotspot of HMW-tau oligomers (Wesseling et al., 2020), was also intensively hyperphosphorylated in AD O-tau (Figure 1C), reflecting heterogeneity in tau phosphorylation among individuals.

AD P-tau has litter activity to promote microtubule assembly and dephosphorylation with AP restored its activity (Alonso et al., 1994). AD P-tau associates with normal tau in solution forming large tangles and dephosphorylation abolishes its ability to aggregate with normal tau and prevents tangle formation (Alonso et al., 1996). Dephosphorylation of AD P-tau by PP2A inhibits its polymerization into PHF/straight filaments, and rephosphorylation of PP2A-dephosphorylated AD P-tau by kinases promotes AD P-tau assembly to PHF filaments (Wang et al., 2007). Intrahippocampal injection of AD P-tau (the same as AD O-tau mentioned in this work) induced tau pathology first at the injection sites, and then to the anatomically connected regions in mouse brain, mimicking the propagation of tau pathology observed in AD patients (Lasagna-Reeves et al., 2012a; Hu et al., 2016). Injection of dephosphorylated AD P-tau treated by protein phosphatase-2A dramatically diminished tau pathology, suggesting that dephosphorylation could restrict the seeding activity of AD P-tau (Hu et al., 2016). However, the mechanisms by which dephosphorylation inhibits the prion-like transmission of tau seeds remain unclear. Our results revealed that dephosphorylation of AD O-tau significantly reduced its ability to capture and template tau aggregation, which may explain why the prion-like seeding activity of dephosphorylated AD O-tau was limited *in vivo*.

AD O-tau and Dp-AD O-tau did not affect the levels of total or phosphorylated tau species in RIPA-soluble cell lysate. AD O-tau induced intensive accumulation of total and phosphorylated tau in the insoluble fraction, while dephosphorylation of AD O-tau significantly blocked that, suggesting that AD O-tau was prone to capture and template phosphorylated tau, and dephosphorylation could effectively inhibit its seeding activity. Therefore, phosphorylation of AD O-tau is crucial for the seeded aggregation of tau, and blockage of AD O-tau phosphorylation could be a potent way to diminish tau aggregation.

Inhibition of tau hyperphosphorylation has long been considered as a potential therapeutic approach for AD. A variety of therapy strategies targets tau hyperphosphorylation by interfering tau kinases or phosphatases (Iqbal et al., 2018). For example, the antidiabetic biguanide metformin induced activation of PP2A and reduced tau phosphorylation at PP2A-dependent epitopes in murine primary neurons and *in vivo* (Kickstein et al., 2010). Recently, a phase II clinical trial for metformin in treating amnestic mild cognitive impairment has been completed. In the present study, we showed that dephosphorylation of AD O-tau by AP, a phosphatase usually located on the outside surface of plasma membrane, significantly inhibited its prion-like seeding activity. AP directly added into the cell cultures could also reduce AD O-tau-induced aggregation of HA-tau_15__1–3__91_ (Figure 4), showing a potential of inhibiting tau pathology. Actually, tissue non-specific alkaline phosphatase (TNAP) activity has been reported to increase in AD brain and plasma (Kellett and Hooper, 2015). Cell membrane-anchored TNAP is capable of dephosphorylating extracellular monomeric tau, which acts as an agonist of muscarinic M1 and M3 receptors, and provokes a sustained intracellular calcium increase leading to neuronal cell death (Diaz-Hernandez et al., 2010; Kellett and Hooper, 2015). Nevertheless, systemic administration of AP is still proposed as a potential therapeutic intervention against the pathology of AD, since sufficient anti-inflammatory AP in circulation might leave endogenous TNAP to perform its normal functions in the brain (Pike et al., 2015). Thus, the role of AP in modulating tau pathology *in vivo* is still worth further investigation.

In conclusion, dephosphorylation significantly decreased AD O-tau’s activity in capturing tau and templating insoluble tau aggregates. Dephosphorylation of AD O-tau could be an effective way to inhibit its prion-like seeding activity and a potential target for the treatment of AD.

## Data Availability Statement

The datasets presented in this article are not readily available because original western blots and immunostaining were used. Requests to access the datasets should be directed to DC, chudd@ntu.edu.cn and FL, fei.liu@opwdd.ny.gov.

## Author Contributions

RW, LL, RS, YZ, NJ, and YT: performing experiments. DC and FL: designing the study, analyzing and interpreting the results, and drafting the manuscript. RW, LL, and FL: critical revision of the manuscript. All authors contributed to the article and approved the submitted version.

## Conflict of Interest

The authors declare that the research was conducted in the absence of any commercial or financial relationships that could be construed as a potential conflict of interest.

## References

[B1] Alonso A. C. Grundke-Iqbal I. Iqbal K. (1996). Alzheimer’s disease hyperphosphorylated tau sequesters normal tau into tangles of filaments and disassembles microtubules. *Nat. Med.* 2 783–787. 10.1038/nm0796-783 8673924

[B2] Alonso A. C. Zaidi T. Grundke-Iqbal I. Iqbal K. (1994). Role of abnormally phosphorylated tau in the breakdown of microtubules in Alzheimer disease. *Proc. Natl. Acad. Sci. U. S. A.* 91 5562–5566. 10.1073/pnas.91.12.5562 8202528 PMC44036

[B3] Austin T. O. Qiang L. Baas P. W. (2017). “Chapter 4 – mechanisms of neuronal microtubule loss in Alzheimer’s disease,” in *Neuroprotection in Alzheimers Disease*, ed. Gozes I. (New York, NY: Sackler School of Medicine), 59–71. 10.1016/b978-0-12-803690-7.00004-1

[B4] Binder L. I. Frankfurter A. Rebhun L. I. (1985). The distribution of tau in the mammalian central nervous system. *J. Cell Biol.* 101 1371–1378. 10.1083/jcb.101.4.1371 3930508 PMC2113928

[B5] Boluda S. Iba M. Zhang B. Raible K. M. Lee V. M. Trojanowski J. Q. (2015). Differential induction and spread of tau pathology in young PS19 tau transgenic mice following intracerebral injections of pathological tau from Alzheimer’s disease or corticobasal degeneration brains. *Acta Neuropathol.* 129 221–237. 10.1007/s00401-014-1373-0 25534024 PMC4305460

[B6] Braak H. Braak E. (1991). Neuropathological stageing of Alzheimer-related changes. *Acta Neuropathol.* 82 239–259. 10.1007/bf00308809 1759558

[B7] Chu D. Liu F. (2018). Pathological changes of Tau related to Alzheimer’s disease. *ACS Chem. Neurosci.* 10 931–944. 10.1021/acschemneuro.8b00457 30346708

[B8] Clavaguera F. Bolmont T. Crowther R. A. Abramowski D. Frank S. Probst A. (2009). Transmission and spreading of tauopathy in transgenic mouse brain. *Nat. Cell Biol.* 11 909–913. 10.1038/ncb1901 19503072 PMC2726961

[B9] Diaz-Hernandez M. Gomez-Ramos A. Rubio A. Gomez-Villafuertes R. Naranjo J. R. Miras-Portugal M. T. (2010). Tissue-nonspecific alkaline phosphatase promotes the neurotoxicity effect of extracellular tau. *J. Biol. Chem.* 285 32539–32548. 10.1074/jbc.m110.145003 20634292 PMC2952256

[B10] Dujardin S. Commins C. Lathuiliere A. Beerepoot P. Fernandes A. R. Kamath T. V. (2020). Tau molecular diversity contributes to clinical heterogeneity in Alzheimer’s disease. *Nat. Med.* 26 1256–1263. 32572268 10.1038/s41591-020-0938-9 PMC7603860

[B11] Goedert M. Jakes R. Spillantini M. G. Hasegawa M. Smith M. J. Crowther R. A. (1996). Assembly of microtubule-associated protein tau into Alzheimer-like filaments induced by sulphated glycosaminoglycans. *Nature* 383 550–553. 10.1038/383550a0 8849730

[B12] Grundke-Iqbal I. Vorbrodt A. W. Iqbal K. Tung Y. C. Wang G. P. Wisniewski H. M. (1988). Microtubule-associated polypeptides tau are altered in Alzheimer paired helical filaments. *Brain Res.* 464 43–52. 10.1016/0169-328x(88)90017-4 3141008

[B13] Gu J. Xu W. Jin N. Li L. Zhou Y. Chu D. (2020). Truncation of tau selectively facilitates its pathological activities. *J. Biol. Chem.* 295 13812–13828. 10.1074/jbc.ra120.012587 32737201 PMC7535906

[B14] Hu W. Zhang X. Tung Y. C. Xie S. Liu F. Iqbal K. (2016). Hyperphosphorylation determines both the spread and the morphology of tau pathology. *Alzheimers Dement.* 12 1066–1077. 10.1016/j.jalz.2016.01.014 27133892

[B15] Iqbal K. Liu F. Gong C. X. (2016). Tau and neurodegenerative disease: the story so far. *Nat. Rev. Neurol.* 12 15–27. 10.1038/nrneurol.2015.225 26635213

[B16] Iqbal K. Liu F. Gong C. X. (2018). Recent developments with tau-based drug discovery. *Expert. Opin. Drug Discov.* 13 399–410. 10.1080/17460441.2018.1445084 29493301

[B17] Kadavath H. Hofele R. V. Biernat J. Kumar S. Tepper K. Urlaub H. (2015). Tau stabilizes microtubules by binding at the interface between tubulin heterodimers. *Proc. Natl. Acad. Sci. U. S. A.* 112 7501–7506. 10.1073/pnas.1504081112 26034266 PMC4475932

[B18] Kellett K. A. Hooper N. M. (2015). The role of tissue non-specific alkaline phosphatase (TNAP) in neurodegenerative diseases: Alzheimer’s disease in the focus. *Subcell. Biochem.* 76 363–374. 10.1007/978-94-017-7197-9_17 26219720

[B19] Kickstein E. Krauss S. Thornhill P. Rutschow D. Zeller R. Sharkey J. (2010). Biguanide metformin acts on tau phosphorylation via mTOR/protein phosphatase 2A (PP2A) signaling. *Proc. Natl. Acad. Sci. U. S. A.* 107 21830–21835. 10.1073/pnas.0912793107 21098287 PMC3003072

[B20] Kopke E. Tung Y. C. Shaikh S. Alonso A. C. Iqbal K. Grundke-Iqbal I. (1993). Microtubule-associated protein tau. Abnormal phosphorylation of a non-paired helical filament pool in Alzheimer disease. *J. Biol. Chem.* 268 24374–24384. 10.1016/s0021-9258(20)80536-5 8226987

[B21] Kremer L. Dominguez J. E. Avila J. (1988). Detection of tubulin-binding proteins by an overlay assay. *Anal. Biochem.* 175 91–95. 10.1016/0003-2697(88)90365-x 3245580

[B22] Lasagna-Reeves C. A. Castillo-Carranza D. L. Sengupta U. Guerrero-Munoz M. J. Kiritoshi T. Neugebauer V. (2012a). Alzheimer brain-derived tau oligomers propagate pathology from endogenous tau. *Sci. Rep.* 2:700. 23050084 10.1038/srep00700 PMC3463004

[B23] Lasagna-Reeves C. A. Castillo-Carranza D. L. Sengupta U. Sarmiento J. Troncoso J. Jackson G. R. (2012b). Identification of oligomers at early stages of tau aggregation in Alzheimer’s disease. *FASEB J.* 26 1946–1959. 10.1096/fj.11-199851 22253473 PMC4046102

[B24] Li L. Jiang Y. Hu W. Tung Y. C. Dai C. Chu D. (2019). Pathological alterations of Tau in Alzheimer’s disease and 3xTg-AD mouse brains. *Mol. Neurobiol.* 56 6168–6183. 10.1007/s12035-019-1507-4 30734228

[B25] Miao J. Shi R. Li L. Chen F. Zhou Y. Tung Y. C. (2019). Pathological Tau from Alzheimer’s brain induces site-specific hyperphosphorylation and SDS- and reducing agent-resistant aggregation of Tau in vivo. *Front. Aging Neurosci.* 11:34. 10.3389/fnagi.2019.00034 30890929 PMC6411797

[B26] Mudher A. Colin M. Dujardin S. Medina M. Dewachter I. Alavi Naini S. M. (2017). What is the evidence that tau pathology spreads through prion-like propagation? *Acta Neuropathol. Commun.* 5:99. 29258615 10.1186/s40478-017-0488-7 PMC5735872

[B27] Pike A. F. Kramer N. I. Blaauboer B. J. Seinen W. Brands R. (2015). An alkaline phosphatase transport mechanism in the pathogenesis of Alzheimer’s disease and neurodegeneration. *Chem. Biol. Interact.* 226 30–39. 10.1016/j.cbi.2014.12.006 25500268

[B28] Planel E. Tatebayashi Y. Miyasaka T. Liu L. Wang L. Herman M. (2007). Insulin dysfunction induces in vivo tau hyperphosphorylation through distinct mechanisms. *J. Neurosci.* 27 13635–13648. 10.1523/jneurosci.3949-07.2007 18077675 PMC6673606

[B29] Takeda S. Wegmann S. Cho H. DeVos S. L. Commins C. Roe A. D. (2015). Neuronal uptake and propagation of a rare phosphorylated high-molecular-weight tau derived from Alzheimer’s disease brain. *Nat. Commun.* 6:8490. 26458742 10.1038/ncomms9490 PMC4608380

[B30] Tatebayashi Y. Iqbal K. Grundke-Iqbal I. (1999). Dynamic regulation of expression and phosphorylation of tau by fibroblast growth factor-2 in neural progenitor cells from adult rat hippocampus. *J. Neurosci.* 19 5245–5254. 10.1523/jneurosci.19-13-05245.1999 10377336 PMC6782310

[B31] von Bergen M. Barghorn S. Biernat J. Mandelkow E. M. Mandelkow E. (2005). Tau aggregation is driven by a transition from random coil to beta sheet structure. *Biochim. Biophys. Acta* 1739 158–166. 10.1016/j.bbadis.2004.09.010 15615635

[B32] Wang J. Z. Grundke-Iqbal I. Iqbal K. (1996). Restoration of biological activity of Alzheimer abnormally phosphorylated tau by dephosphorylation with protein phosphatase-2A, -2B and -1. *Brain Res. Mol. Brain Res.* 38 200–208. 10.1016/0169-328x(95)00316-k 8793108

[B33] Wang J. Z. Grundke-Iqbal I. Iqbal K. (2007). Kinases and phosphatases and tau sites involved in Alzheimer neurofibrillary degeneration. *Eur. J. Neurosci.* 25 59–68. 10.1111/j.1460-9568.2006.05226.x 17241267 PMC3191918

[B34] Wang Y. Garg S. Mandelkow E. M. Mandelkow E. (2010). Proteolytic processing of tau. *Biochem. Soc. Trans.* 38 955–961. 10.1042/bst0380955 20658984

[B35] Weingarten M. D. Lockwood A. H. Hwo S. Y. Kirschner M. W. (1975). A protein factor essential for microtubule assembly. *Proc. Natl. Acad. Sci. U. S. A.* 72 1858–1862. 10.1073/pnas.72.5.1858 1057175 PMC432646

[B36] Wesseling H. Mair W. Kumar M. Schlaffner C. N. Tang S. Beerepoot P. (2020). Tau PTM profiles identify patient heterogeneity and stages of Alzheimer’s disease. *Cell* 183:e13. 10.1016/j.cell.2020.10.029 PMC8168922 33188775

[B37] Wischik C. M. Schelter B. O. Wischik D. J. Storey J. M. D. Harrington C. R. (2018). Modeling prion-like processing of Tau protein in Alzheimer’s disease for pharmaceutical development. *J. Alzheimers Dis.* 62 1287–1303. 10.3233/jad-170727 29226873 PMC5870021

[B38] Zhang Z. Song M. Liu X. Kang S. S. Kwon I. S. Duong D. M. (2014). Cleavage of tau by asparagine endopeptidase mediates the neurofibrillary pathology in Alzheimer’s disease. *Nat. Med.* 20 1254–1262. 10.1038/nm.3700 25326800 PMC4224595

[B39] Zhou Y. Shi J. Chu D. Hu W. Guan Z. Gong C. X. (2018). Relevance of phosphorylation and truncation of Tau to the etiopathogenesis of Alzheimer’s disease. *Front. Aging Neurosci.* 10:27. 10.3389/fnagi.2018.00027 29472853 PMC5810298

